# Is End-Stage Ankle Arthrosis Best Managed with Total Ankle Replacement or Arthrodesis? A Systematic Review

**DOI:** 10.1155/2014/986285

**Published:** 2014-08-21

**Authors:** Robert W. Jordan, Gurdip S. Chahal, Anna Chapman

**Affiliations:** ^1^University Hospitals Coventry & Warwickshire, Clifford Bridge Road, Coventry CV2 2DX, UK; ^2^Birmingham Heartlands Hospital, Bordesley Green East, Birmingham B9 5SS, UK

## Abstract

*Introduction*. End-stage ankle osteoarthritis is a debilitating condition. Traditionally, ankle arthrodesis (AA) has been the surgical intervention of choice but the emergence of total ankle replacement (TAR) has challenged this concept. This systematic review aims to address whether TAR or AA is optimal in terms of functional outcomes. *Methods*. We conducted a systematic review according to PRISMA checklist using the online databases Medline and EMBASE after January 1, 2005. Participants must be skeletally mature and suffering from ankle arthrosis of any cause. The intervention had to be an uncemented TAR comprising two or three modular components. The comparative group could include any type of ankle arthrodesis, either open or arthroscopic, using any implant for fixation. The study must have reported at least one functional outcome measure. *Results*. Of the four studies included, two reported some significant improvement in functional outcome in favour of TAR. The complication rate was higher in the TAR group. However, the quality of studies reviewed was poor and the methodological weaknesses limited any definitive conclusions being drawn. *Conclusion*. The available literature is insufficient to conclude which treatment is superior. Further research is indicated and should be in the form of an adequately powered randomised controlled trial.

## 1. Introduction

End-stage ankle osteoarthritis is a debilitating condition that results in functional limitations and a poor quality of life [1]. The incidence of symptomatic osteoarthritis of the ankle has been estimated to be 47.7/100,000 in the United Kingdom [2]. More than 70% is related to posttraumatic osteoarthritis [1, 3] with the majority of the remainder being primary arthritis, inflammatory arthritis, or secondary to osteonecrosis. Mild to moderate ankle arthritis can often be managed with ankle foot orthoses and a rocker-bottom shoe [4]. When surgical intervention is indicated, ankle arthrodesis (AA) has traditionally been used. However, the development of total ankle replacement (TAR) has challenged arthrodesis as the treatment of choice for ankle arthrosis [5–7].

AA has been shown to give good results in multiple papers [8–10]. Various techniques have been used which include cannulated screws, plate fixation, retrograde nail, and external fixation. The main drawback of the procedure is the high rate of later arthrosis in adjacent joints, reported between 10 and 60% [11–13]. This results in a subsequent subtalar fusion rate at 5 years of 2.8% which is higher than the 0.7% after TAR [14]. Additional complications associated with AA include wound infection (3–25%), nonunion (10–20%), and malalignment [15, 16].

TAR is still evolving [17] but has the potential advantage to preserve range of motion, restore gait, and, thus, protect adjacent articulations [18]. The results from first generation ankle prosthesis were disappointing with stiffness, wound complications, loosening, malalignment, impingement, and instability cited as causes of failure [7, 19–21]. Newer prosthesis is uncemented, comprises two or three modular components, and has improved outcomes. A recent systematic review reported 89% survival of 7942 TARs at 10 years and an improvement in American Orthopaedic Foot and Ankle Society score (AOFAS) from 40 to 80 at a mean of 8.2 years of follow-up [22]. However, a further review from Gougoulias et al. of 1105 total ankle arthroplasties reported a 10% failure rate at 5 years with residual pain in 27%–60% of cases [23].

Haddad et al. performed a systematic review of the literature up until 2005 comparing outcomes following second generation TAR and AA. However, the majority of the 49 studies reviewed were single centre case series and none directly compared the two treatments. They concluded that both techniques gave comparable intermediate and long-term outcomes, reporting good or excellent outcomes in 68.5% of TAR and 67% of AA patients and mean AOFAS scores of 78.2 following TAR and 75.6 following AA. However, variability of reporting outcomes and lack of controlled studies restricted direct comparisons of the two groups [24]. The aim of this paper is to perform an up-to-date systematic review of the literature including only comparative studies addressing whether TAR or AA is optimal in the treatment of end-stage ankle arthrosis in terms of functional outcomes.

## 2. Methods

We conducted a systematic review of the literature using the online databases Medline and EMBASE. The review was performed and reported according to the PRISMA checklist. The search strategy used for the Medline search is shown in Table 1 and this was modified for searching EMBASE. The searches were carried out on January 15, 2014, and limited to papers available in English. The search was limited to papers published after January 1, 2005, as the previous systematic review published in 2007 included papers prior to this date [24].

Inclusion criteria were applied. Participants must be skeletally mature and suffering from ankle arthrosis of any cause deemed severe enough to warrant either TAR or AA by the treating surgeon. The intervention had to be an uncemented TAR comprising two or three modular components. The comparative group could include any type of ankle arthrodesis, either open or arthroscopic, using any implant for fixation. The study must have reported at least one functional outcome measure. Studies were excluded if no comparative group was analysed or if the study participants were divided into more than two groups. In addition, only primary research was considered for review with any abstracts, comments, review articles, and technique articles excluded. Eligibility of studies was assessed independently by two authors (R.J. and G.C.) who also appraised the included studies against the STROBE statement [25]. If there was any disagreement between the authors in assigning a score to each paper appraised, a third independent reviewer (A.C.) made the final decision.

## 3. Results

**Figure 1:**
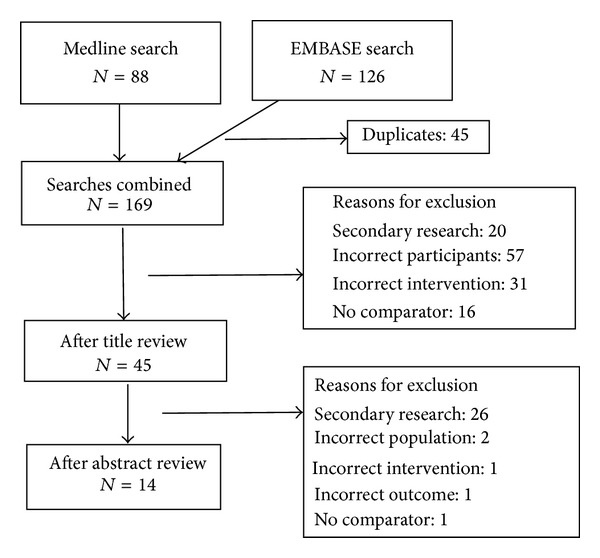
Flow diagram of review process.

The Medline search revealed 88 and the EMBASE search 126 results.  Figure 1 shows a flow diagram of the review process including the reasons for exclusion at different stages of the process. Concise details of the included studies are given in Table 2 and the appraisal against STROBE statement [25] is given in Table 3. The reasons for exclusion of the 10 articles at full paper review stage are given in Table 4.

Of the four studies included, three were level III retrospective comparative studies and the other was a level II prospective comparative study. The optimal study design when comparing two treatment modalities is a randomised controlled trial (RCT) as this allows for minimisation of bias. The main limitation that is common to all four studies reviewed is the lack of randomisation; this risks selection bias with the uneven allocation of confounding factors between the groups. In addition, as none of the studies reviewed defined a primary outcome measure or included a power calculation, uncertainty is present as to whether any of the studies was sufficiently powered to show a significant difference in any recorded outcome measure.

*Schuh et al., 2012.* Schuh et al. [26] performed a retrospective review of 63 patients that underwent either TAR, using a HINTEGRA prosthesis (Newdeal SA, Lyon, France), or AA, using 3 cannulated screws. No significant difference was found between the two groups in terms of activity level, participation in sport, or American Orthopaedic Foot and Ankle Society (AOFAS) hindfoot score. The surgery was performed by a single, fellowship trained surgeon between 1998 and 2006. Of the 63 patients assessed, only 41 were included for analysis. The 16% loss to follow-up forms a high proportion and the majority (70%) of these were in the AA group. The reason these patients refused follow-up is not known and their exclusion may have skewed results. Minimal details of the inclusion criteria are stated and whether patients with arthrosis of any cause were included is not clear. Clarity is required to decide whether results are applicable to other populations. In addition, no mention is given as to whether assessments were performed by an independent observer or the postoperative physiotherapy regimen was consistent between the two groups.

*Esparragoza et al., 2011.* Esparragoza et al. [27] performed a prospective comparative study of 30 patients. Surgery was performed by a total of 6 senior staff members of whom 3 performed all of the TAR and 3 all of the AA. All patients undergoing TAR had the Ankle Evolution System implant (Biomet, Nimes, France) inserted but arthrodesis was performed by three different techniques. A statistically significant improvement in AOFAS (*P* = 0.048) and SF-36 (*P* = 0.026) in the TAR group was reported at two years. However, the study had a number of limitations including a lack of randomisation and inclusion of only a small number of patients. Although the reported baseline characteristics are similar between the groups, further information on adjacent joint arthrosis would have been beneficial. Those patients undergoing arthrodesis were further divided into three techniques, which introduces heterogeneity amongst this arthrodesis group. Provision of outcomes for each technique would have demonstrated whether all techniques had equal effectiveness but the low number of patients studied precluded this. Again, details regarding the postoperative physiotherapy regime and those responsible for measuring outcomes should have been provided.

*Krause et al., 2011.* Krause et al. [28] performed a retrospective comparative study of 161 patients. No statistically significant difference in functional outcome was reported between the groups; however, TAR patients had a higher complication rate (*P* = 0.003). Clear indications for each procedure are set out and details regarding the surgical technique, surgeon experience, postoperative regime, and definitions of terms are given. Due to strict inclusion and exclusion criteria, only 31% of patients were deemed eligible to participate limiting the external validity of results. The authors excluded cases where a failed arthrodesis was converted to a TAR but included cases where a failed TAR was converted to AA. Conversion procedures are likely to be more complex and so, prone to worse results; the inclusion of failed TAR but not failed AA potentially increases the complexity of cases in the AA group and this may have impacted outcomes. The ankle replacement used as the intervention could be one of four prostheses depending on surgeon preference: the Agility ankle system (Depuy, Warsaw, IN, USA), the Mobility ankle system (Depuy, Warsaw, IN, USA), Scandinavian Total Ankle Replacement (STAR) (Waldmar Link, Hamburg, Germany), and HINTEGRA prosthesis (Newdeal SA, Lyon, France). This is a pragmatic approach but the outcomes of different prostheses may vary and this is not accounted for in the study. Comparison between the two groups is limited by their differences at baseline and complexity of surgery performed. The fusion group was younger, had a lower proportion of rheumatoid arthritis, and had a much higher proportion of low complexity surgery (87% versus 32%); any positive results may result from these differences rather than the type of surgery received.

*Saltzman et al., 2010.* Saltzman et al. [29] performed a retrospective comparative study of 71 patients undergoing either TAR or AA, using a STAR (Waldmar Link, Hamburg, Germany). A significant improvement in the TAR group in the pain component of Ankle Osteoarthritis Scale (AOS) (*P* = 0.001) and the mental component of SF-36 (*P* = 0.011) was reported. Initially, 138 patients were assessed for eligibility but 67 did not satisfy inclusion criteria and this high number questions the external validity of results. Of the 71 included, 11 were lost to follow-up, with slightly more lost in the AA group. Patients undergoing TAR with an alternative prosthesis were excluded; in contrast, those undergoing AA had three different surgical techniques performed. This approach is slightly contradictory as there is an assumption that patients having differing TAR replacements may have varying outcomes but those having various arthrodeses have equal outcomes. A pragmatic approach would be to compare all replacements versus all arthrodeses, or alternatively to compare one replacement against one fusion technique. At the beginning of this section, the importance of randomisation and transparency of treatment allocation was discussed. In the current study, the AA group had a higher number of young patients, males, and those with posttraumatic arthritis. Each of these factors may directly influence outcome and thus any positive results cannot be confidently attributed to the treatment given. Outcome measures have only been recorded postoperatively and the lack of preoperative value limits the value of the results as the degree of improvement following treatment is not known.

## 4. Discussion

Four studies were identified and reviewed which addressed our research question. Two of the four studies reported statistically significant improvements in functional outcomes following TAR [27, 29]; the other two studies showed no differences between the two groups [26, 28]. However, the methodological flaws present stop definitive conclusions being drawn.

The main limitation in design common to all studies was the lack of randomisation. This risks differences in the study groups being present at the point of treatment allocation which has the potential to affect results. Only one study described the indications for the two procedures [28], using severe deformity or instability, poor ankle motion, no or mild adjacent joint arthritis, and younger age as indications of arthrodesis. Therefore, the arthrodesis group in this study will have had a higher proportion of patients with these factors than the TAR group, which all may have influenced outcomes. The other three studies [26, 27, 29] do not describe the indications used for allocation but it is likely that baseline characteristics also differed in these studies, limiting the ability to directly compare outcomes between the two groups. A further limitation of the systematic review is the use of pragmatic entry criteria for the intervention and comparator groups, with all arthrodesis procedures and any uncemented ankle replacement included. The four studies included used five different uncemented ankle prostheses and four fixation methods for arthrodesis including open and arthroscopic techniques. The inclusion of numerous surgical techniques restricts the generalisability of the results to any specific technique as it is likely that the outcomes following each method differ.

Although at least one functional outcome was measured in each study, the evidence supporting the validity, reliability, and responsiveness of the available measures following foot and ankle surgery is limited [30, 31]. The AOFAS is the most commonly used outcome measure following foot and ankle surgery [30, 31] but it relies on the observer to measure both range of motion and malalignment, risking observer bias. The AOS is patient reported which reduces the risk of observer bias and has been validated in ankle osteoarthritis, but it is not validated in the measurement of outcome following AA or TAR. Therefore, neither of the two functional measures provides an ideal measurement tool and may have contributed to inaccurate recording of outcomes. Future research should utilise an outcome tool that both is patient reported and is validated for use in this postoperative population.

At present, the evidence is insufficient to change clinical practice. Arthrodesis has been the traditional treatment of choice and will continue to be so until the literature definitively demonstrates one modality to be superior to the other. The increased rate of complications reported following TAR further supports this approach [28]. Ideally, future research will be of RCT design so that selection bias is limited and effects measured can be attributed to treatment allocation. The population treated needs to be clearly defined so that results can be applied to the readers' practice. They should have a patient reported outcome as the defined primary outcome and the study should be adequately powered to show a statistically significant difference between the groups.

## 5. Conclusion

Although half of the reviewed studies report some functional improvement following total ankle replacement, the lack of high quality evidence limits a definitive conclusion being drawn. Insufficient evidence is available to decide whether total ankle replacement or ankle arthrodesis improves functional outcomes and further research in the form of robust RCTs is indicated.

## Figures and Tables

**Table 1:** Search strategy for Medline.

Number	Search term	Results
1	exp Ankle Joint/or exp Ankle/	16259
2	exp Arthroplasty, Replacement/or exp Arthroplasty/or exp Arthroplasty, Replacement, Ankle/	37169
3	exp Arthrodesis/	22930
4	1 and 2 and 3	141
5	limit 4 to (english language and humans)	128
6	limit 5 to yr = “2005–Current”	88

**Table 2:** Summary of the included studies.

Study	Study Design	Population	Intervention	Comparator	Outcome measures (primary in bold)	Follow-up period	Significant results
Schuh et al., 2012 [26] (*n* = 41)	Retrospective comparative study—level III	Adults with ankle osteoarthritis who have failed conservative treatment	HINTEGRA prosthesis—uncemented 3-component TAR (*n* = 20)	Arthrodesis with 3 ∗ cannulated screws (*n* = 21)	Patient satisfaction Sports and activity questionnaire Halasi ankle score UCLA activity score AOFAS	34.5 months	No significant results

Esparragoza et al., 2011 [27] (*n* = 30)	Prospective comparative study—level II	Adult primary or secondary ankle arthritis	Ankle Evolution System prosthesis—uncemented 3-component TAR (*n* = 14)	Open arthrodesis with bone graft and either external fixator, screw fixation, or retrograde calcaneal nail (*n* = 16)	AOFAS SF-36 Complications	24 months	Improved results in TAR group: AOFAS (*P* = 0.048) SF-36 (*P* = 0.026)

Krause et al., 2011 [28] (*n* = 161)	Retrospective comparative study—level III	Adults with primary or secondary arthritis, revision of TAR to AA included	TAR with one of four uncemented prostheses: agility (two components), mobility (three components), STAR, HINTEGRA (*n* = 114)	Open or arthroscopic arthrodesis (*n* = 47)	AOS COFAS classification Complications Radiographic	Mean 37 months	Higher complication rate with TAR 54% versus 26% (*P* = 0.003) Higher severity of COFAS in TAR group

Saltzman et al., 2010 [29] (*n* = 71)	Retrospective comparative study—level III	Adult posttraumatic or primary ankle osteoarthritis	STAR prosthesis—uncemented 3-component TAR (*n* = 42)	Open arthrodesis with external fixator, plate fixation, or screw fixation (*n* = 29)	AOS SF-36 Complications Pre and postoperative Radiographic	4.2 years	Improved results in TAR group: Pain component AOS (*P* = 0.001) SF-36 mental component (*P* = 0.011) Higher complication in TAR 15/37 versus 5/23 AA

**Table 3:** Assessment of papers according to STROBE statement [24].

	Item number	Recommendation	Schuh et al., 2012 [26]	Esparragoza et al., 2011 [27]	Saltzman et al., 2010 [29]	Krause et al., 2011 [28]
Title and abstract	1	(a) Indicate the study's design with a commonly used term in the title or the abstract.	No	Yes	No	No
		(b) Provide in the abstract an informative and balanced summary of what was done and what was found.	Yes	Yes	Yes	Yes

Introduction						
Background/rationale	2	Explain the scientific background and rationale for the investigation being reported.	Yes	Yes	Yes	Yes
Objectives	3	State specific objectives, including any prespecified hypotheses.	Yes	Yes	Yes	Yes

Methods						
Study design	4	Present key elements of study design early in the paper.	No	Yes	Yes	Yes
Setting	5	Describe the setting, locations, and relevant dates, including periods of recruitment, exposure, follow-up, and data collection.	Yes	Yes	Yes	Yes
Participants	6	(a) *Cohort study*—give the eligibility criteria and the sources and methods of selection of participants. Describe methods of follow-up. *Case-control study*—give the eligibility criteria and the sources and methods of case ascertainment and control selection. Give the rationale for the choice of cases and controls. *Cross-sectional study*—give the eligibility criteria and the sources and methods of selection of participants.	No	No	No	Yes
		(b) *Cohort study*—for matched studies, give matching criteria and number of exposed and unexposed. *Case-control study*—for matched studies, give matching criteria and the number of controls per case.	n/a	n/a	n/a	n/a
Variables	7	Clearly define all outcomes, exposures, predictors, potential confounders, and effect modifiers. Give diagnostic criteria, if applicable.	Yes	Yes	Yes	Yes
Data sources/measurement	8	For each variable of interest, give sources of data and details of methods of assessment (measurement). Describe comparability of assessment methods if there is more than one group.	No	No	No	Yes
Bias	9	Describe any efforts to address potential sources of bias.	No	No	No	Yes
Study size	10	Explain how the study size was arrived at.	No	No	No	No
Quantitative variables	11	Explain how quantitative variables were handled in the analyses. If applicable, describe which groupings were chosen and why.	Yes	No	Yes	Yes
Statistical methods	12	(a) Describe all statistical methods, including those used to control for confounding.	Yes	Yes	Yes	Yes
		(b) Describe any methods used to examine subgroups and interactions.	n/a	n/a	n/a	n/a
		(c) Explain how missing data were addressed.	No	n/a	No	No
		(d) *Cohort study*—if applicable, explain how loss to follow-up was addressed. *Case-control study*—if applicable, explain how matching of cases and controls was addressed. *Cross-sectional study*—if applicable, describe analytical methods taking account of sampling strategy.	No	n/a	No	n/a
		(e) Describe any sensitivity analyses.	n/a	n/a	n/a	n/a
	13	(a) Report numbers of individuals at each stage of study—for example, numbers of potentially eligible, examined for eligibility, confirmed eligible, included in the study, completing follow-up, and analysed.	No	No	Yes	Yes
		(b) Give reasons for nonparticipation at each stage.	No	n/a	Yes	Yes
		(c) Consider use of a flow diagram.	No	No	No	No
Descriptive data	14	(a) Give characteristics of study participants (e.g., demographic, clinical, and social) and information on exposures and potential confounders.	No	Yes	Yes	Yes
		(b) Indicate number of participants with missing data for each variable of interest.	No	No	No	No
		(c) *Cohort study*—summarise follow-up time (e.g., average and total amount).	Yes	Yes	Yes	
Outcome data	15	*Cohort study*—report numbers of outcome events or summary measures over time.	No	No	No	No
		*Case-control study—*report numbers in each exposure category, or summary measures of exposure.				
		*Cross-sectional study—*report numbers of outcome events or summary measures.				
Main results	16	(a) Give unadjusted estimates and, if applicable, confounder-adjusted estimates and their precision (e.g., 95% confidence interval). Make clear which confounders were adjusted for and why they were included.	No	No	No	No
		(b) Report category boundaries when continuous variables were categorized.	n/a	n/a	n/a	n/a
		(c) If relevant, consider translating estimates of relative risk into absolute risk for a meaningful time period.	n/a	n/a	n/a	n/a
Other analyses	17	Report other analyses done—for example, analyses of subgroups and interactions and sensitivity analyses.	n/a	n/a	n/a	n/a

Discussion						
Key results	18	Summarise key results with reference to study objectives.	Yes	Yes	Yes	Yes
Limitations	19	Discuss limitations of the study, taking into account sources of potential bias or imprecision. Discuss both direction and magnitude of any potential bias.	Yes	No	Yes	Yes
Interpretation	20	Give a cautious overall interpretation of results considering objectives, limitations, multiplicity of analyses, results from similar studies, and other relevant lines of evidence.	Yes	No	Yes	Yes
Generalisability	21	Discuss the generalisability (external validity) of the study results.	No	No	No	No

Other information						
Funding	22	Give the source of funding and the role of the funders in the present study and, if applicable, in the original study on which the present paper is based.	No	No	No	Yes

**Table 4:** Reason for exclusion of studies after full paper review.

Study	Reason for exclusion
[32]	Expert opinion
Conley et al., 2012 [33]	No functional outcome recorded
Flavin et al., 2013 [34]	No functional outcome recorded
Hahn et al., 2012 [35]	No functional outcome recorded
Krause and Schmid 2012 [36]	No functional outcome recorded
Piriou et al., 2008 [18]	No functional outcome recorded
Rouhani et at., 2011 [37]	Study participants divided into more than two groups
Rouhani et al., 2012 [38]	Study participants divided into more than two groups
Slobogean et al., 2010 [39]	No functional outcome recorded
SooHoo et al., 2007 [14]	No functional outcome recorded

## References

[1] Saltzman CL, Zimmerman MB, O'Rourke M, Brown TD, Buckwalter JA, Johnston R (2006). Impact of comorbidities on the measurement of health in patients with ankle osteoarthritis. *Journal of Bone and Joint Surgery A*.

[2] Goldberg AJ, MacGregor A, Dawson J (2012). The demand incidence of symptomatic ankle osteoarthritis presenting to foot & ankle surgeons in the United Kingdom. *Foot*.

[3] Thomas R, Daniels TR, Parker K (2006). Gait analysis and functional outcomes following ankle arthrodesis for isolated ankle arthritis. *Journal of Bone and Joint Surgery A*.

[4] Martin RL, Stewart GW, Conti SF (2007). Posttraumatic ankle arthritis: an update on conservative and surgical management. *Journal of Orthopaedic and Sports Physical Therapy*.

[5] Pyevich MT, Saltzman CL, Callaghan JJ, Alvine FG (1998). Total ankle arthroplasty: a unique design: two to twelve-year follow-up. *Journal of Bone and Joint Surgery A*.

[6] Anderson T, Montgomery F, Carlsson A (2003). Uncemented STAR total ankle prostheses: three to eight-year follow-up of fifty-one consecutive ankles. *Journal of Bone and Joint Surgery A*.

[7] Kofoed H, Sørensen TS (1998). Ankle arthroplasty for rheumatoid arthritis and osteoarthritis. *Journal of Bone and Joint Surgery B*.

[8] Chen Y, Huang T, Shih H, Hsu K, Hsu RW (1996). Ankle arthrodesis with cross-screw fixation. Good results in 36/40 cases followed 3–7 years. *Acta Orthopaedica Scandinavica*.

[9] Holt ES, Hansen ST, Mayo KA, Sangeorzan BJ (1991). Ankle arthrodesis using internal screw fixation. *Clinical Orthopaedics and Related Research*.

[10] Thomas RH, Daniels TR (2003). Ankle arthritis. *Journal of Bone and Joint Surgery A*.

[11] Coester LM, Saltzman CL, Leupold J, Pontarelli W (2001). Long-term results following ankle arthrodesis for post-traumatic arthritis. *Journal of Bone and Joint Surgery A*.

[12] Fuchs S, Sandmann C, Skwara A, Chylarecki C (2003). Quality of life 20 years arthrodesis of the ankle. *Journal of Bone and Joint Surgery B*.

[13] Morrey BF, Wideman GP (1980). Complications and long-term results of ankle arthrodeses following trauma. *Journal of Bone and Joint Surgery A*.

[14] SooHoo NF, Zingmond DS, Ko CY (2007). Comparison of reoperation rates following ankle arthrodesis and total ankle arthroplasty. *Journal of Bone and Joint Surgery - Series A*.

[15] Bauer G, Kinzl L (1996). Arthrodesis of the ankle joint. *Orthopade*.

[16] Cooper PS (2001). Complications of ankle and tibiotalocalcaneal arthrodesis. *Clinical Orthopaedics and Related Research*.

[17] Culpan P, Le Strat V, Piriou P, Judet T (2007). Arthrodesis after failed total ankle replacement. *Journal of Bone and Joint Surgery - Series B*.

[18] Piriou P, Culpan P, Mullins M, Cardon JN, Pozzi D, Judet T (2008). Ankle replacement versus arthrodesis: a comparative gait analysis study. *Foot and Ankle International*.

[19] Newton SE (1982). Total ankle arthroplasty. Clinical study of fifty cases. *Journal of Bone and Joint Surgery A*.

[20] Bolton-Maggs BG, Sudlow RA, Freeman MAR (1985). Total ankle arthroplasty: a long-term review of the London hospital experience. *Journal of Bone and Joint Surgery B*.

[21] Kitaoka HB, Patzer GL, Ilstrup DM, Wallrichs SL (1994). Survivorship analysis of the Mayo total ankle arthroplasty. *Journal of Bone and Joint Surgery A*.

[22] Zaidi R, Cro S, Gurusamy K (2013). The outcome of total ankle replacement: a systematic review and meta-analysis. *The Bone & Joint Journal*.

[23] Gougoulias N, Khanna A, Maffulli N (2010). How successful are current ankle replacements?: a systematic review of the literature. *Clinical Orthopaedics and Related Research*.

[24] Haddad SL, Coetzee JC, Estok R, Fahrbach K, Banel D, Nalysnyk L (2007). Intermediate and long-term outcomes of total ankle arthroplasty and ankle arthrodesis: a systematic review of the literature. *Journal of Bone and Joint Surgery A*.

[25] STROBE statement http://www.strobe-statement.org/fileadmin/Strobe/uploads/checklists/STROBE_checklist_v4_combined.pdf.

[26] Schuh R, Hofstaetter J, Krismer M, Bevoni R, Windhager R, Trnka H (2012). Total ankle arthroplasty versus ankle arthrodesis. Comparison of sports, recreational activities and functional outcome. *International Orthopaedics*.

[27] Esparragoza L, Vidal C, Vaquero J (2011). Comparative study of the quality of life between arthrodesis and total arthroplasty substitution of the ankle. *Journal of Foot and Ankle Surgery*.

[28] Krause FG, Windolf M, Bora B, Penner MJ, Wing KJ, Younger ASE (2011). Impact of complications in total ankle replacement and ankle arthrodesis analyzed with a validated outcome measurement. *Journal of Bone and Joint Surgery A*.

[29] Saltzman CL, Kadoko RG, Suh JS (2010). Treatment of isolated ankle osteoarthritis with arthrodesis or the total ankle replacement: a comparison of early outcomes. *Clinics in Orthopedic Surgery*.

[30] Naal FD, Impellizzeri FM, Rippstein PF (2010). Which are the most frequently used outcome instruments in studies on total ankle arthroplasty?. *Clinical Orthopaedics and Related Research*.

[31] Hunt KJ, Hurwit D (2013). Use of patient-reported outcome measures in foot and ankle research. *Journal of Bone and Joint Surgery A*.

[32] (2012). Ankle replacement tops ankle fusion as new gold standard. The procedure has evolved into come in, have surgery, go home the next day. *Duke Medicine Health News*.

[33] Conley KA, Geist K, Shaw JN, Labib SA, Johanson MA (2012). The effect of goniometric alignment on passive ankle dorsiflexion range of motion among patients following ankle arthrodesis or arthroplasty. *Foot and Ankle Specialist*.

[34] Flavin R, Coleman SC, Tenenbaum S, Brodsky JW (2013). Comparison of gait after total ankle arthroplasty and ankle arthrodesis. *Foot & Ankle International*.

[35] Hahn ME, Wright ES, Segal AD, Orendurff MS, Ledoux WR, Sangeorzan BJ (2012). Comparative gait analysis of ankle arthrodesis and arthroplasty: initial findings of a prospective study. *Foot and Ankle International*.

[36] Krause FG, Schmid T (2012). Ankle arthrodesis versus total ankle replacement. How do I decide?. *Foot & Ankle Clinics*.

[37] Rouhani H, Crevoisier X, Favre J, Aminian K (2011). Outcome evaluation of ankle osteoarthritis treatments: plantar pressure analysis during relatively long-distance walking. *Clinical Biomechanics*.

[38] Rouhani H, Favre J, Aminian K, Crevoisier X (2012). Multi-segment foot kinematics after total ankle replacement and ankle arthrodesis during relatively long-distance gait. *Gait and Posture*.

[39] Slobogean GP, Younger A, Apostle KL (2010). Preference-based quality of life of end-stage ankle arthritis treated with arthroplasty or arthrodesis. *Foot and Ankle International*.

